# Crystal structures of 1,4-di­aza­bicyclo­[2.2.2]octan-1-ium 4-nitro­benzoate dihydrate and 1,4-di­aza­bicyclo­[2.2.2]octane-1,4-diium bis­(4-nitro­benzoate): the influence of solvent upon the stoichiometry of the formed salt

**DOI:** 10.1107/S1600536814011532

**Published:** 2014-06-23

**Authors:** Aina Mardia Akhmad Aznan, Zanariah Abdullah, Edward R. T. Tiekink

**Affiliations:** a Department of Chemistry, University of Malaya, 50603 Kuala Lumpur, Malaysia

**Keywords:** crystal structure, amine, carb­oxy­lic acid, co-crystallization, salt formation

## Abstract

The 1:1 co-crystallization of 1,4-di­aza­bicyclo­[2.2.2]octane (DABCO) with 4-nitro­benzoic acid in ethanol–water (3/1) gave the salt dihydrate C_6_H_13_N_2_^+^·C_7_H_4_NO_4_^−^·2H_2_O, (1), whereas from methanol, the salt C_6_H_14_N_2_^2+^·2C_7_H_4_NO_4_^−^, (2), was isolated. In (1), the cation and anion are linked by a strong N—H⋯O hydrogen bond, and the carboxyl­ate anion is close to planar [dihedral angle between terminal residues = 6.83 (9)°]. In (2), a three-ion aggregate is assembled by two N—H⋯O hydrogen bonds, and the carboxyl­ate anions are again close to planar [dihedral angles between terminal residues = 1.7 (3) and 5.9 (3)°]. Through the inter­vention of solvent water mol­ecules, which self-assemble into helical supra­molecular chains along the *b* axis, the three-dimensional architecture in (1) is stabilized by water–DABCO O—H⋯N and water–carboxyl­ate O—H⋯O hydrogen bonds, with additional stability afforded by C—H⋯O inter­actions. The global crystal structure comprises alternating layers of water mol­ecules and ion pairs stacked along the *c* axis. In the crystal of (2), the three-ion aggregates are assembled into a three-dimensional architecture by a large number of methyl­ene–carboxyl­ate/nitro C—H⋯O inter­actions as well as π–π contacts between inversion-related benzene rings [inter-centroid distances = 3.5644 (16) and 3.6527 (16) Å]. The cations and anions assemble into alternating layers along the *c* axis.

## Abstract 2

Solvent-dependent outcomes are noted in co-crystallization experiments between DABCO and 4-nitrobenzoic acid with mono- and diprotonated forms of DABCO are isolated.

## Chemical context   

The formation of co-crystals or salts is dependent on the difference in p*K_a_* of the inter­acting species (Childs *et al.*, 2007 ▸). Thus, when the Δ(p*K_a_*) [= p*K_a_*(base) − p*K_a_*(acid)] value is greater than three, a salt is anti­cipated. In this context, it is not surprising that a search of the Cambridge Structural Database (CSD, version 53.5, last update November 2013; Allen, 2002 ▸) showed that nearly 90% of the 57 multi-component crystals, containing species derived from highly basic 1,4-di­aza­bicyclo­[2.2.2]octane (DABCO) and a carb­oxy­lic acid, contained at least a mono-protonated form of DABCO. It was in the context of on-going studies of co-crystallization experiments (Broker & Tiekink, 2007 ▸; Arman & Tiekink, 2013 ▸; Arman *et al.*, 2014 ▸) between nitro­gen-containing mol­ecules and carb­oxy­lic acids, that the title salts were isolated. The co-crystallization experiments yielding the title salts produced unexpected outcomes in that while (1) formed as a 1:1 salt dihydrate from the 1:1 co-crystallization of DABCO and 4-nitro­benzoic acid in ethanol/water (3/1) solution, a 1:2 salt (2) was isolated from the 1:1 co-crystallization of DABCO and 4-nitro­benzoic acid in methanol solution. The mol­ecular and crystal structures of (1) and (2) are described herein.
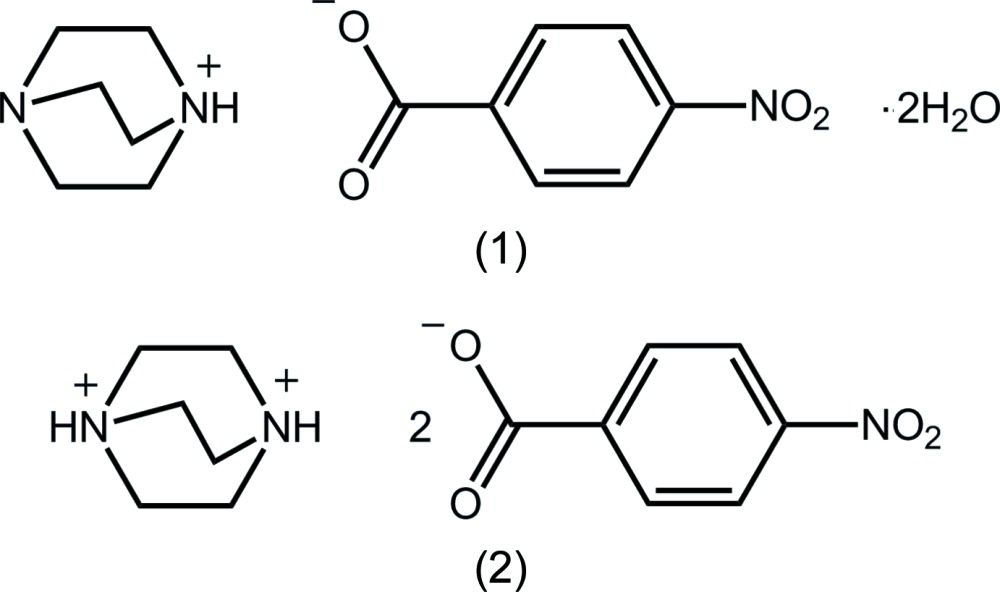


## Structural commentary   

**Figure 1:**
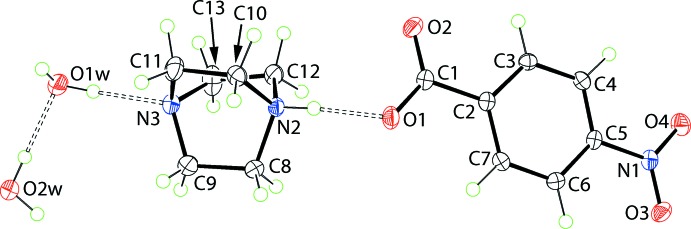
The mol­ecular structures of the four independent constituents of (1), with atom labelling. Displacement ellipsoids are drawn at the 50% probability level. Hydrogen bonds are shown as dashed lines (see Table 1 ▸ for details).

The asymmetric unit of (1) comprises a 1,4-di­aza­bicyclo­[2.2.2]octan-1-ium mono-cation, a 4-nitro­benzoate anion and two water mol­ecules of hydration (Fig. 1 ▸). The most notable feature in the cation is the elongation of the N2—C bond lengths [1.4951 (16)–1.5007 (15) Å] compared to the N3—C bond lengths [1.4635 (17)–1.4773 (17) Å], consistent with protonation at the N2 atom. In the anion, the near equivalence of the C1—O1,O2 bond lengths of 1.2625 (15) and 1.2495 (16) Å, respectively, is again consistent with proton transfer; the longer bond involves atom O1 which forms a strong N—H⋯O hydrogen bond (Table 1 ▸). The dihedral angles between the central ring and the carboxyl­ate and nitro groups are 8.68 (8) and 3.80 (5)°, respectively, and the dihedral angle between the terminal groups is 6.83 (9)°, consistent with an approximately planar mol­ecule.

**Figure 2:**
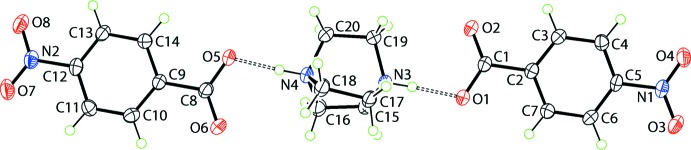
The mol­ecular structures of the three independent constituents of (2), with atom labelling. Displacement ellipsoids are drawn at the 50% probability level. Hydrogen bonds are shown as dashed lines (see Table 2 ▸ for details).

The asymmetric unit of (2) comprises a 1,4-di­aza­bicyclo­[2.2.2]octane-1,4-diium di-cation and two 4-nitro­benzoate anions (Fig. 2 ▸). In the dication, the N3—C [1.483 (3)–1.487 (3) Å] and N4—C [1.486 (3)–1.487 (3) Å] bond lengths are experimentally equivalent and consistent with diprotonation. In the anions, the disparity of the C1—O1, O2 bond lengths, *i.e*. 1.281 (3) and 1.228 (3) Å, is slightly greater than that in C8—O5, O6 of 1.273 (3) and 1.231 (3) Å, respectively. In each case the longer bond forms a strong N—H⋯O hydrogen bond (Table 2 ▸). In order to confirm the location of the acidic hydrogen atoms, an unrestrained refinement was conducted, see *Refinement* for details. While there was some elongation in the N—H bond lengths, unrestrained refinement confirmed protonation at both nitro­gen atoms. In the O1-containing anion, the dihedral angles between the central ring and the carboxyl­ate and nitro groups are 7.0 (3) and 8.7 (2)°, respectively, and the dihedral angle between the terminal groups is 1.7 (3)°. The comparable angles for the O5-containing anion are 2.2 (3), 7.4 (2) and 5.9 (3)°, respectively. As discussed below in *Supra­molecular features*, the ions participate in strong N—H⋯O hydrogen bonds, forming a three-ion aggregate (Fig. 2 ▸) in which the dihedral angle between the benzene rings is 9.26 (14)°.

## Supra­molecular features   

**Figure 3:**
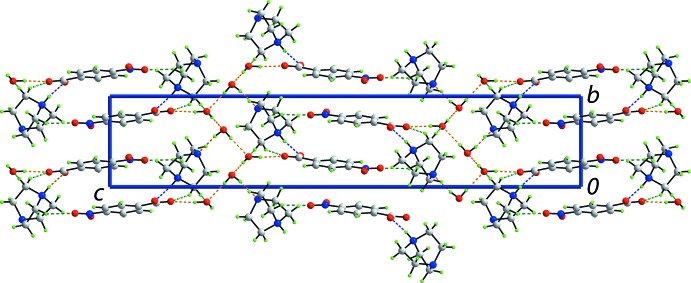
Unit-cell contents shown in projection down the *a* axis for (1). The O—H⋯O, O—H⋯N and C—H⋯O hydrogen bonds are shown as orange, blue and green dashed lines, respectively (see Table 1 ▸ for details).

In (1), the cation and anion are linked by a strong N2—H⋯O1 hydrogen bond (Fig. 1 ▸ and Table 1 ▸). The two ion aggregates inter-digitate in columns aligned along the *b* axis. Adjacent columns stack along the *a* axis to form layers in the *ab* plane. The layers are inter­spersed by layers of water mol­ecules which self-assemble into helical chains along the *b* axis, where each independent water mol­ecule donates and accepts a water-O—H⋯O(water) hydrogen bond (Table 1 ▸). This leads to the formation of a three-dimensional structure (Fig. 3 ▸).

Thus, links between layers are of the type water-O1*W*—H⋯N3 and water-O*W*2—H⋯O2(carboxyl­ate). Additional stability to the supra­molecular assembly is afforded by methyl­ene-C—H⋯O2(carboxyl­ate) and O2*W*(water) inter­actions; it is noteworthy that both of the former inter­actions involve hydrogen atoms derived from the same methyl­ene-C12 atom (Table 1 ▸). A methyl­ene-C—H⋯O3(nitro) inter­action is also formed; the nitro-O4 atom does not form a significant inter­action in this scenario. Although there is an alignment of benzene rings, the closest π–π contact is 3.7376 (7) Å, occurring between centrosymmetrically related rings [symmetry operation: 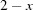, 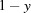, 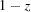].

**Figure 4:**
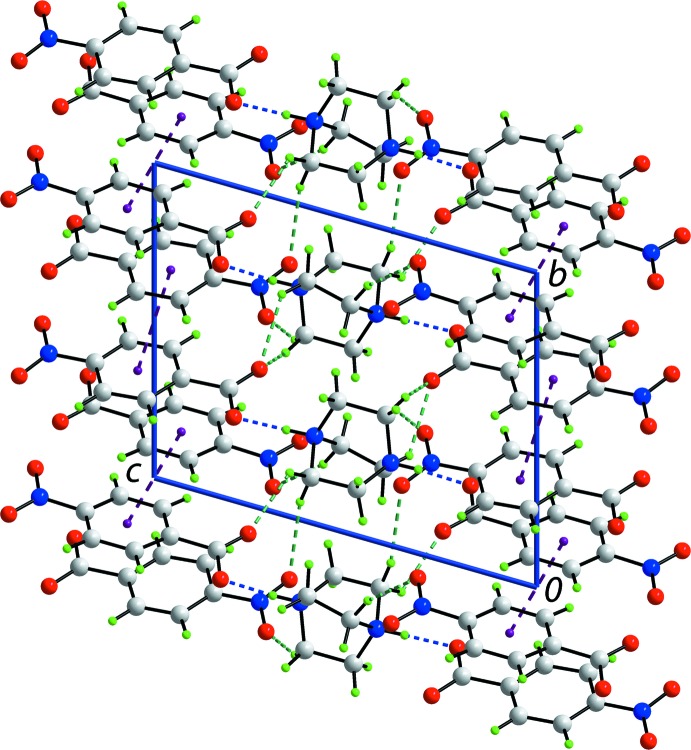
Unit-cell contents shown in projection down the *a* axis for (2). The N—H⋯O and C—H⋯O hydrogen bonds are shown as blue and green dashed lines, respectively (see Table 2 ▸ for details).

**Figure 5:**
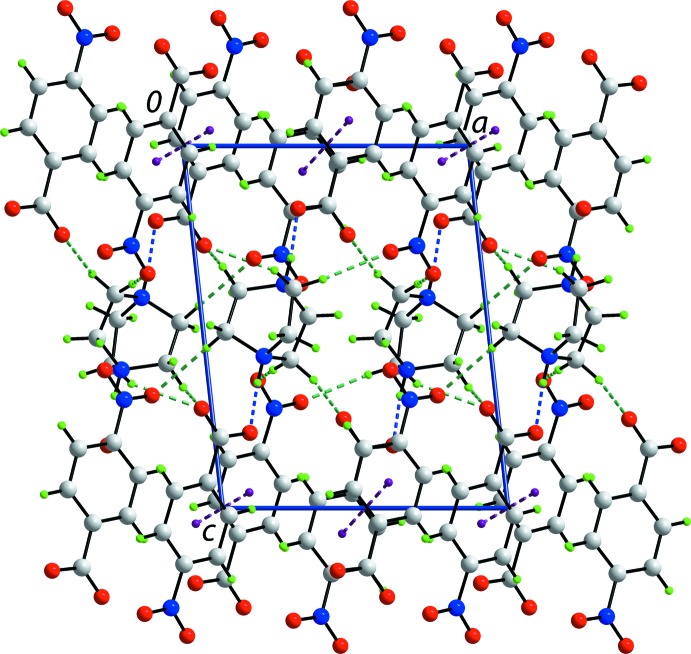
Unit-cell contents shown in projection down the *b* axis for (2). The N—H⋯O and C—H⋯O hydrogen bonds are shown as blue and green dashed lines, respectively (see Table 2 ▸ for details).

In (2), the di-cation is linked to two anions *via* strong N—H⋯O hydrogen bonds (Fig. 4 ▸ and Table 2 ▸). Globally, the three ion aggregates assemble into layers in the *ab* plane that stack along the *c* axis. A large number of C—H⋯O inter­actions occur, remarkably featuring a narrow range of H⋯O separations, *i.e*. 2.41–2.42 Å (Table 2 ▸). All inter­actions involve methyl­ene-H atoms as donors. The carboxyl­ate-O2 and O4 atoms and all nitro but O3 atoms are acceptors; both methyl­ene-H atoms of methyl­ene-C17 and C20 participate in C—H⋯O inter­actions. The result of these inter­actions is the formation of a three-dimensional architecture (Fig. 4 ▸). Additional stability to the supra­molecular assembly is afforded by π—π inter­actions between inversion-related rings, *i.e*. inter-centroid distances = 3.5644 (16) Å for inter­actions between the C2–C8 rings (symmetry code: −*x* + 1, −*y* + 2, −*z* + 2) and 3.6527 (16) Å between C9–C14 rings (symmetry code: −*x* + 2, −*y* + 1, −*z*). An alternate description of the global crystal packing is based on alternating of layers of cations and layers of anions along the *c* axis (Fig. 5 ▸).

## Database survey   

As mentioned in the *Chemical context*, there are 57 species in the crystallographic literature containing DABCO or its mono- or diprotonated forms and a carb­oxy­lic acid or carboxyl­ate anion. In fact, co-crystals are rare, being around 10% of all structures. Co-crystals are formed with several di­carb­oxy­lic acids where the functional groups are separated by long chains of over four carbon atoms (Braga *et al.*, 2003 ▸; Moon & Park, 2012 ▸), with phosphono­acetic acid (Bowes *et al.*, 2003 ▸) and with isophthalic acid (Marivel *et al.*, 2010 ▸). While the majority of the remaining structures contain species derived from a di­carb­oxy­lic acid, there are 13 examples of structures containing species derived from a mono-carb­oxy­lic acid which are more directly suitable for comparison with (1) and (2). Further, in each case the original carb­oxy­lic acid was connected to an aromatic ring. Of the sub-set of 13 structures, three are similar to (1), having the mono-protonated form of DABCO. The carboxyl­ate counter-ions are 2,4-di­nitro­benzoate (Rosli *et al.*, 2006 ▸), 3,5-di­hydroxy­benzoate (Burchell *et al.*, 2001*a* ▸) and 6-hy­droxy-2-naphtho­ate (Jacobs *et al.*, 2010 ▸); the latter two structures were characterized as mono- and sesqui-hydrates, respectively. Analogues of (2) were found in seven examples, namely in both polymorphs of benzoate, and in 2-hy­droxy­benzoate and 2-acet­oxy­benzoate (Skovsgaard & Bond, 2009 ▸), 2-chloro­benzoate (Skovsgaard & Bond, 2008 ▸), 2-hy­droxy­benzoate (Skovsgaard & Bond, 2008 ▸), and in polymorphic hydrates of 3,5-di­nitro­benzoate (Burchell *et al.*, 2001*b* ▸; Chantrapromma & Fun, 2004 ▸). Finally, there are three intriguing examples where a mono-protonated DABCO cation is present along with a carboxyl­ate anion and the neutral form of the original carb­oxy­lic acid. These contain the following carb­oxy­lic acids: 1-hy­droxy-2-naphthoic acid and 3-hy­droxy-2-naphthoic acid (Jacobs *et al.*, 2010 ▸) and 2-amino­benzoic acid (Arman *et al.*, 2011 ▸). In light of the foregoing structural diversity, in retrospect perhaps it is not so surprising that solvent can influence product formation, especially when water is involved.

## Synthesis and crystallization   

1,4-Di­aza­bicyclo­[2.2.2]octane (Merck; 0.10 g, 0.0009 mol), was mixed with 4-nitro­benzoic acid (Merck; 0.15 g, 0.0009 mol) in a solution containing ethanol (30 ml) and water (10 ml). The solution was heated for 2 h at 350 K. The mixture was then left for slow evaporation and colourless crystals of (1) formed after 4 days. In a similar experiment, 1,4-di­aza­bicyclo­[2.2.2]octane (0.298 g, 0.00265 mol) was mixed with 4-nitro­benzoic acid (0.444 g, 0.00265 mol) in a solution of methanol (50 ml). The solution was heated for 2 h at 345 K. The mixture was then left for slow evaporation and colourless crystals of (2) formed after 4 days.

## Refinement   

Crystal data, data collection and structure refinement details are summarized in Table 3 ▸. Carbon-bound H-atoms were placed in calculated positions (C—H = 0.95–0.99 Å) and were included in the refinement in the riding-model approximation, with *U*_iso_(H) = 1.2*U*_eq_(C). The N-bound H-atoms were located in a difference Fourier map but were refined with a distance restraint: N—H = 0.88 (1) Å with *U*_iso_(H) = 1.2*U*_eq_(N). For (1), the water-bound H atoms were refined with distance restraints: O—H = 0.84 (1) and H⋯H = 1.39 (2) Å with *U*_iso_(H) =1.5*U*_eq_(O). For (2), the maximum and minimum residual electron density peaks of 0.60 and 0.58 e Å^−3^, respectively, were located 0.81 and 0.10 Å from atoms O5 and H4*N*, respectively. In order to confirm the location of the N-bound H atoms, in a separate refinement these were refined without distance restraints. For (1), the N2—H2*N* bond length was 0.948 (17) Å. For (2), the N3—H3*N* and N4—H4*N* bond lengths were 0.93 (4) and 1.08 (3) Å, respectively. In the refinement of (1), one reflection, *i.e*. (180), was omitted from the refinement owing to poor agreement. For the same reasons, the following reflections were omitted from the final refinement of (2): (550), (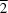
06), (136), (139), (2410), (331), (224) and (662).

## Supplementary Material

Crystal structure: contains datablock(s) 1, 2, global. DOI: 10.1107/S1600536814011532/su0005sup1.cif

Structure factors: contains datablock(s) 1. DOI: 10.1107/S1600536814011532/su00051sup2.hkl

Structure factors: contains datablock(s) 2. DOI: 10.1107/S1600536814011532/su00052sup3.hkl

Supporting information file. DOI: 10.1107/S1600536814011532/su00051sup4.cml

Supporting information file. DOI: 10.1107/S1600536814011532/su00052sup5.cml

CCDC references: 1004283, 1004284

Additional supporting information:  crystallographic information; 3D view; checkCIF report

## Figures and Tables

**Table 1:** Hydrogen-bond geometry (Å, °) for (1)

*D*—H⋯*A*	*D*—H	H⋯*A*	*D*⋯*A*	*D*—H⋯*A*
N2—H2*N*⋯O1	0.89 (1)	1.76 (1)	2.6431 (14)	173 (1)
O1*W*—H1*W*⋯N3	0.86 (2)	1.95 (2)	2.7974 (15)	172 (2)
O1*W*—H2*W*⋯O2*W* ^i^	0.86 (2)	1.91 (1)	2.7500 (15)	165 (2)
O2*W*—H3*W*⋯O1*W*	0.85 (2)	1.87 (2)	2.7218 (15)	180 (2)
O2*W*—H4*W*⋯O2^ii^	0.86 (1)	1.87 (1)	2.7182 (15)	171 (2)
C10—H10*A*⋯O3^iii^	0.99	2.49	3.4253 (17)	158
C12—H12*A*⋯O2*W* ^iv^	0.99	2.49	3.3711 (16)	147
C12—H12*B*⋯O2^v^	0.99	2.57	3.4818 (16)	153

Symmetry codes: (i) 
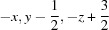
; (ii) 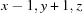; (iii) 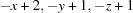; (iv) 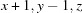; (v) 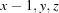.

**Table 2:** Hydrogen-bond geometry (Å, °) for (2)

*D*—H⋯*A*	*D*—H	H⋯*A*	*D*⋯*A*	*D*—H⋯*A*
N3—H3*N*⋯O1	0.88 (2)	1.66 (2)	2.539 (3)	173 (2)
N4—H4*N*⋯O5	0.89 (2)	1.65 (2)	2.542 (3)	175 (3)
C15—H15*A*⋯O6^i^	0.99	2.42	3.193 (3)	134
C16—H16*A*⋯O4^ii^	0.99	2.42	3.375 (3)	161
C17—H17*A*⋯O7^iii^	0.99	2.42	3.338 (3)	153
C17—H17*B*⋯O6^i^	0.99	2.42	3.313 (3)	149
C20—H20*A*⋯O2^iv^	0.99	2.41	3.043 (3)	121
C20—H20*B*⋯O8^v^	0.99	2.42	3.339 (3)	154

Symmetry codes: (i) 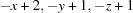; (ii) 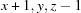; (iii) 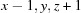; (iv) 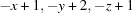; (v) 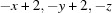.

**Table 3:** Experimental details

	(1)	(2)
Crystal data		
Chemical formula	C_6_H_13_N_2_^+^·C_7_H_4_NO_4_^−^·2H_2_O	C_6_H_14_N_2_^2+^·2C_7_H_4_NO_4_^−^
*M*_r_	315.33	446.42
Crystal system, space group	Monoclinic, *P*2_1_/*c*	Triclinic, *P* 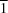
Temperature (K)	100	100
*a*, *b*, *c* (Å)	6.5982 (1), 6.6074 (1), 34.4574 (6)	9.1036 (4), 9.5027 (3), 12.0736 (3)
α, β, γ (°)	90, 94.809 (1), 90	73.982 (3), 83.624 (3), 88.661 (3)
*V* (Å^3^)	1496.95 (4)	997.68 (6)
*Z*	4	2
Radiation type	Cu *K*α	Cu *K*α
μ (mm^−1^)	0.94	0.99
Crystal size (mm)	0.30 × 0.30 × 0.20	0.40 × 0.40 × 0.20

Data collection		
Diffractometer	Agilent SuperNova Dual with an Atlas detector	Agilent SuperNova Dual with an Atlas detector
Absorption correction	Multi-scan (*CrysAlis PRO*; Agilent, 2013 ▸)	Multi-scan (*CrysAlis PRO*; Agilent, 2013 ▸)
*T*_min_, *T*_max_	0.888, 1.000	0.991, 1.000
No. of measured, independent and observed [*I* > 2σ(*I*)] reflections	11203, 3072, 2949	17849, 4101, 3775
*R*_int_	0.015	0.046
(sin θ/λ)_max_ (Å^−1^)	0.626	0.626

Refinement		
*R*[*F* ^2^ > 2σ(*F* ^2^)], *wR*(*F* ^2^), *S*	0.039, 0.099, 1.04	0.074, 0.229, 1.12
No. of reflections	3072	4101
No. of parameters	215	295
No. of restraints	7	2
H-atom treatment	H atoms treated by a mixture of independent and constrained refinement	H atoms treated by a mixture of independent and constrained refinement
Δρ_max_, Δρ_min_ (e Å^−3^)	0.40, −0.30	0.60, −0.58

Computer programs: *CrysAlis PRO* (Agilent, 2013 ▸), *SHELXS97* and *SHELXL97* (Sheldrick, 2008 ▸), *ORTEP-3 for Windows* (Farrugia, 2012 ▸), *DIAMOND* (Brandenburg, 2006 ▸) and *publCIF* (Westrip, 2010 ▸).

## supplementary crystallographic information

### (1) 1,4-Diazabicyclo[2.2.2]octan-1-ium 4-nitrobenzoate dihydrate Crystal data

**Table:**

C_6_H_13_N_2_^+^·C_7_H_4_NO_4_^−^·2H_2_O	*F*(000) = 672
*M**_r_* = 315.33	*D*_x_ = 1.399 Mg m^−^^3^
Monoclinic, *P*2_1_/*c*	Cu *K*α radiation, λ = 1.54184 Å
Hall symbol: -P 2ybc	Cell parameters from 7046 reflections
*a* = 6.5982 (1) Å	θ = 3.9–76.3°
*b* = 6.6074 (1) Å	µ = 0.94 mm^−^^1^
*c* = 34.4574 (6) Å	*T* = 100 K
β = 94.809 (1)°	Prism, colourless
*V* = 1496.95 (4) Å^3^	0.30 × 0.30 × 0.20 mm
*Z* = 4	

### (1) 1,4-Diazabicyclo[2.2.2]octan-1-ium 4-nitrobenzoate dihydrate Data collection

**Table:**

Agilent SuperNova Dual diffractometer with an Atlas detector	3072 independent reflections
Radiation source: SuperNova (Cu) X-ray Source	2949 reflections with *I* > 2σ(*I*)
Mirror monochromator	*R*_int_ = 0.015
Detector resolution: 10.4041 pixels mm^-1^	θ_max_ = 75.0°, θ_min_ = 5.2°
ω scan	*h* = −8→8
Absorption correction: multi-scan (CrysAlis PRO; Agilent, 2013)	*k* = −8→5
*T*_min_ = 0.888, *T*_max_ = 1.000	*l* = −43→42
11203 measured reflections	

### (1) 1,4-Diazabicyclo[2.2.2]octan-1-ium 4-nitrobenzoate dihydrate Refinement

**Table:**

Refinement on *F*^2^	Secondary atom site location: difference Fourier map
Least-squares matrix: full	Hydrogen site location: inferred from neighbouring sites
*R*[*F*^2^ > 2σ(*F*^2^)] = 0.039	H atoms treated by a mixture of independent and constrained refinement
*wR*(*F*^2^) = 0.099	*w* = 1/[σ^2^(*F*_o_^2^) + (0.0467*P*)^2^ + 0.8811*P*] where *P* = (*F*_o_^2^ + 2*F*_c_^2^)/3
*S* = 1.04	(Δ/σ)_max_ = 0.001
3072 reflections	Δρ_max_ = 0.40 e Å^−^^3^
215 parameters	Δρ_min_ = −0.30 e Å^−^^3^
7 restraints	Extinction correction: SHELXL97 (Sheldrick, 2008), Fc^*^=kFc[1+0.001xFc^2^λ^3^/sin(2θ)]^-1/4^
Primary atom site location: structure-invariant direct methods	Extinction coefficient: 0.0076 (6)

### (1) 1,4-Diazabicyclo[2.2.2]octan-1-ium 4-nitrobenzoate dihydrate Special details

**Table:**

Geometry. All s.u.'s (except the s.u. in the dihedral angle between two l.s. planes) are estimated using the full covariance matrix. The cell s.u.'s are taken into account individually in the estimation of s.u.'s in distances, angles and torsion angles; correlations between s.u.'s in cell parameters are only used when they are defined by crystal symmetry. An approximate (isotropic) treatment of cell s.u.'s is used for estimating s.u.'s involving l.s. planes.
Refinement. Refinement of *F*^2^ against ALL reflections. The weighted *R*-factor *wR* and goodness of fit *S* are based on *F*^2^, conventional *R*-factors *R* are based on *F*, with *F* set to zero for negative *F*^2^. The threshold expression of *F*^2^ > 2σ(*F*^2^) is used only for calculating *R*-factors(gt) *etc*. and is not relevant to the choice of reflections for refinement. *R*-factors based on *F*^2^ are statistically about twice as large as those based on *F*, and *R*- factors based on ALL data will be even larger.

### (1) 1,4-Diazabicyclo[2.2.2]octan-1-ium 4-nitrobenzoate dihydrate Fractional atomic coordinates and isotropic or equivalent isotropic displacement parameters (Å^2^)

**Table:**

	*x*	*y*	*z*	*U*_iso_*/*U*_eq_	
O1	0.63866 (13)	0.34327 (15)	0.59514 (3)	0.0233 (2)	
O2	0.94425 (14)	0.33794 (16)	0.62902 (3)	0.0261 (2)	
O3	1.11387 (14)	0.20664 (15)	0.42606 (3)	0.0234 (2)	
O4	1.40402 (14)	0.17440 (16)	0.45978 (3)	0.0262 (2)	
N1	1.21908 (16)	0.20014 (15)	0.45723 (3)	0.0175 (2)	
C1	0.82971 (19)	0.32425 (18)	0.59836 (4)	0.0178 (3)	
C2	0.93105 (18)	0.28490 (18)	0.56096 (3)	0.0153 (2)	
C3	1.13841 (18)	0.24078 (18)	0.56251 (3)	0.0162 (3)	
H3	1.2149	0.2318	0.5871	0.019*	
C4	1.23420 (18)	0.20989 (18)	0.52871 (4)	0.0163 (2)	
H4	1.3751	0.1786	0.5297	0.020*	
C5	1.11798 (18)	0.22606 (17)	0.49335 (3)	0.0149 (2)	
C6	0.91145 (18)	0.26736 (17)	0.49062 (3)	0.0155 (2)	
H6	0.8354	0.2752	0.4660	0.019*	
C7	0.81863 (17)	0.29702 (18)	0.52492 (3)	0.0156 (2)	
H7	0.6772	0.3258	0.5238	0.019*	
N2	0.48292 (15)	0.59677 (17)	0.64400 (3)	0.0178 (2)	
H2N	0.535 (2)	0.503 (2)	0.6291 (4)	0.021*	
N3	0.33337 (16)	0.86385 (17)	0.68589 (3)	0.0207 (2)	
C8	0.31552 (19)	0.7058 (2)	0.62050 (4)	0.0213 (3)	
H8A	0.2168	0.6074	0.6082	0.026*	
H8B	0.3720	0.7863	0.5997	0.026*	
C9	0.2099 (2)	0.8456 (2)	0.64826 (4)	0.0309 (3)	
H9A	0.1903	0.9811	0.6363	0.037*	
H9B	0.0744	0.7900	0.6527	0.037*	
C10	0.64491 (19)	0.7447 (2)	0.65768 (4)	0.0212 (3)	
H10A	0.7121	0.7988	0.6352	0.025*	
H10B	0.7491	0.6777	0.6756	0.025*	
C11	0.5423 (2)	0.9174 (2)	0.67881 (5)	0.0325 (3)	
H11A	0.6212	0.9456	0.7039	0.039*	
H11B	0.5416	1.0418	0.6628	0.039*	
C12	0.39871 (18)	0.49677 (19)	0.67830 (4)	0.0199 (3)	
H12A	0.5038	0.4108	0.6923	0.024*	
H12B	0.2810	0.4105	0.6695	0.024*	
C13	0.3323 (2)	0.6654 (2)	0.70520 (4)	0.0257 (3)	
H13A	0.1938	0.6361	0.7127	0.031*	
H13B	0.4257	0.6688	0.7292	0.031*	
O1W	0.19593 (16)	1.12932 (16)	0.74123 (3)	0.0268 (2)	
H1W	0.243 (3)	1.058 (3)	0.7232 (4)	0.040*	
H2W	0.151 (3)	1.039 (2)	0.7565 (4)	0.040*	
O2W	−0.12706 (15)	1.33213 (17)	0.70557 (3)	0.0289 (2)	
H3W	−0.026 (2)	1.268 (3)	0.7167 (5)	0.043*	
H4W	−0.117 (3)	1.328 (3)	0.6810 (3)	0.043*	

### (1) 1,4-Diazabicyclo[2.2.2]octan-1-ium 4-nitrobenzoate dihydrate Atomic displacement parameters (Å^2^)

**Table:**

	*U*^11^	*U*^22^	*U*^33^	*U*^12^	*U*^13^	*U*^23^
O1	0.0175 (4)	0.0299 (5)	0.0231 (5)	0.0006 (4)	0.0055 (3)	−0.0078 (4)
O2	0.0235 (5)	0.0391 (6)	0.0158 (4)	0.0014 (4)	0.0025 (4)	−0.0027 (4)
O3	0.0271 (5)	0.0281 (5)	0.0153 (4)	0.0000 (4)	0.0033 (4)	0.0001 (4)
O4	0.0181 (5)	0.0355 (6)	0.0262 (5)	0.0027 (4)	0.0087 (4)	0.0009 (4)
N1	0.0200 (5)	0.0147 (5)	0.0185 (5)	−0.0011 (4)	0.0058 (4)	0.0004 (4)
C1	0.0190 (6)	0.0167 (6)	0.0181 (6)	−0.0009 (5)	0.0042 (5)	−0.0018 (5)
C2	0.0170 (6)	0.0122 (5)	0.0171 (6)	−0.0017 (4)	0.0034 (4)	−0.0006 (4)
C3	0.0165 (6)	0.0154 (6)	0.0165 (6)	−0.0012 (4)	−0.0004 (4)	0.0003 (4)
C4	0.0137 (5)	0.0144 (5)	0.0209 (6)	−0.0006 (4)	0.0026 (4)	0.0007 (4)
C5	0.0178 (6)	0.0116 (5)	0.0159 (6)	−0.0020 (4)	0.0053 (4)	−0.0006 (4)
C6	0.0174 (6)	0.0124 (5)	0.0164 (5)	−0.0022 (4)	−0.0002 (4)	0.0002 (4)
C7	0.0135 (5)	0.0133 (5)	0.0200 (6)	−0.0016 (4)	0.0021 (4)	−0.0005 (4)
N2	0.0148 (5)	0.0219 (5)	0.0171 (5)	0.0002 (4)	0.0033 (4)	−0.0034 (4)
N3	0.0212 (5)	0.0228 (6)	0.0187 (5)	0.0016 (4)	0.0044 (4)	−0.0023 (4)
C8	0.0200 (6)	0.0270 (7)	0.0167 (6)	0.0018 (5)	0.0005 (5)	−0.0011 (5)
C9	0.0331 (7)	0.0375 (8)	0.0217 (7)	0.0148 (6)	−0.0002 (6)	−0.0017 (6)
C10	0.0153 (6)	0.0274 (7)	0.0215 (6)	−0.0047 (5)	0.0041 (5)	−0.0018 (5)
C11	0.0276 (7)	0.0300 (7)	0.0416 (8)	−0.0100 (6)	0.0126 (6)	−0.0138 (7)
C12	0.0174 (6)	0.0207 (6)	0.0219 (6)	−0.0009 (5)	0.0039 (5)	0.0027 (5)
C13	0.0299 (7)	0.0274 (7)	0.0210 (6)	0.0011 (5)	0.0104 (5)	0.0017 (5)
O1W	0.0311 (5)	0.0285 (5)	0.0210 (5)	0.0053 (4)	0.0026 (4)	−0.0024 (4)
O2W	0.0258 (5)	0.0420 (6)	0.0191 (5)	0.0084 (4)	0.0041 (4)	0.0005 (4)

### (1) 1,4-Diazabicyclo[2.2.2]octan-1-ium 4-nitrobenzoate dihydrate Geometric parameters (Å, º)

**Table:**

O1—C1	1.2625 (15)	N3—C13	1.4706 (17)
O2—C1	1.2495 (16)	N3—C9	1.4773 (17)
O3—N1	1.2298 (14)	C8—C9	1.5381 (18)
O4—N1	1.2279 (14)	C8—H8A	0.9900
N1—C5	1.4704 (15)	C8—H8B	0.9900
C1—C2	1.5231 (16)	C9—H9A	0.9900
C2—C7	1.3943 (17)	C9—H9B	0.9900
C2—C3	1.3956 (16)	C10—C11	1.5408 (19)
C3—C4	1.3857 (17)	C10—H10A	0.9900
C3—H3	0.9500	C10—H10B	0.9900
C4—C5	1.3884 (17)	C11—H11A	0.9900
C4—H4	0.9500	C11—H11B	0.9900
C5—C6	1.3852 (17)	C12—C13	1.5365 (18)
C6—C7	1.3900 (16)	C12—H12A	0.9900
C6—H6	0.9500	C12—H12B	0.9900
C7—H7	0.9500	C13—H13A	0.9900
N2—C10	1.4951 (16)	C13—H13B	0.9900
N2—C8	1.4982 (16)	O1W—H1W	0.861 (9)
N2—C12	1.5007 (15)	O1W—H2W	0.866 (9)
N2—H2N	0.892 (9)	O2W—H3W	0.852 (9)
N3—C11	1.4635 (17)	O2W—H4W	0.856 (9)
			
O4—N1—O3	123.50 (10)	N2—C8—H8B	110.2
O4—N1—C5	118.32 (10)	C9—C8—H8B	110.2
O3—N1—C5	118.18 (10)	H8A—C8—H8B	108.5
O2—C1—O1	126.58 (11)	N3—C9—C8	110.47 (11)
O2—C1—C2	116.76 (11)	N3—C9—H9A	109.6
O1—C1—C2	116.65 (11)	C8—C9—H9A	109.6
C7—C2—C3	119.51 (11)	N3—C9—H9B	109.6
C7—C2—C1	120.35 (11)	C8—C9—H9B	109.6
C3—C2—C1	120.13 (11)	H9A—C9—H9B	108.1
C4—C3—C2	120.90 (11)	N2—C10—C11	107.60 (10)
C4—C3—H3	119.6	N2—C10—H10A	110.2
C2—C3—H3	119.6	C11—C10—H10A	110.2
C3—C4—C5	117.96 (11)	N2—C10—H10B	110.2
C3—C4—H4	121.0	C11—C10—H10B	110.2
C5—C4—H4	121.0	H10A—C10—H10B	108.5
C6—C5—C4	122.86 (11)	N3—C11—C10	110.91 (11)
C6—C5—N1	118.62 (11)	N3—C11—H11A	109.5
C4—C5—N1	118.52 (10)	C10—C11—H11A	109.5
C5—C6—C7	118.10 (11)	N3—C11—H11B	109.5
C5—C6—H6	120.9	C10—C11—H11B	109.5
C7—C6—H6	120.9	H11A—C11—H11B	108.0
C6—C7—C2	120.66 (11)	N2—C12—C13	107.39 (10)
C6—C7—H7	119.7	N2—C12—H12A	110.2
C2—C7—H7	119.7	C13—C12—H12A	110.2
C10—N2—C8	109.35 (10)	N2—C12—H12B	110.2
C10—N2—C12	109.99 (9)	C13—C12—H12B	110.2
C8—N2—C12	109.42 (9)	H12A—C12—H12B	108.5
C10—N2—H2N	109.7 (10)	N3—C13—C12	111.17 (10)
C8—N2—H2N	109.0 (10)	N3—C13—H13A	109.4
C12—N2—H2N	109.4 (11)	C12—C13—H13A	109.4
C11—N3—C13	109.35 (11)	N3—C13—H13B	109.4
C11—N3—C9	109.36 (11)	C12—C13—H13B	109.4
C13—N3—C9	107.53 (11)	H13A—C13—H13B	108.0
N2—C8—C9	107.72 (10)	H1W—O1W—H2W	103.0 (15)
N2—C8—H8A	110.2	H3W—O2W—H4W	107.8 (16)
C9—C8—H8A	110.2		
			
O2—C1—C2—C7	−170.56 (11)	C1—C2—C7—C6	178.03 (11)
O1—C1—C2—C7	8.23 (17)	C10—N2—C8—C9	−67.76 (13)
O2—C1—C2—C3	8.02 (17)	C12—N2—C8—C9	52.77 (14)
O1—C1—C2—C3	−173.19 (11)	C11—N3—C9—C8	50.98 (16)
C7—C2—C3—C4	0.32 (18)	C13—N3—C9—C8	−67.65 (14)
C1—C2—C3—C4	−178.27 (11)	N2—C8—C9—N3	13.17 (16)
C2—C3—C4—C5	0.59 (18)	C8—N2—C10—C11	53.58 (13)
C3—C4—C5—C6	−1.34 (18)	C12—N2—C10—C11	−66.60 (13)
C3—C4—C5—N1	178.05 (10)	C13—N3—C11—C10	51.74 (15)
O4—N1—C5—C6	176.15 (11)	C9—N3—C11—C10	−65.75 (15)
O3—N1—C5—C6	−3.43 (16)	N2—C10—C11—N3	11.63 (16)
O4—N1—C5—C4	−3.27 (16)	C10—N2—C12—C13	53.46 (13)
O3—N1—C5—C4	177.15 (10)	C8—N2—C12—C13	−66.68 (13)
C4—C5—C6—C7	1.12 (18)	C11—N3—C13—C12	−65.51 (14)
N1—C5—C6—C7	−178.28 (10)	C9—N3—C13—C12	53.13 (14)
C5—C6—C7—C2	−0.14 (17)	N2—C12—C13—N3	11.20 (14)
C3—C2—C7—C6	−0.56 (18)		

### (1) 1,4-Diazabicyclo[2.2.2]octan-1-ium 4-nitrobenzoate dihydrate Hydrogen-bond geometry (Å, º)

**Table:**

*D*—H···*A*	*D*—H	H···*A*	*D*···*A*	*D*—H···*A*
N2—H2*N*···O1	0.89 (1)	1.76 (1)	2.6431 (14)	173 (1)
O1*W*—H1*W*···N3	0.86 (2)	1.95 (2)	2.7974 (15)	172 (2)
O1*W*—H2*W*···O2*W*^i^	0.86 (2)	1.91 (1)	2.7500 (15)	165 (2)
O2*W*—H3*W*···O1*W*	0.85 (2)	1.87 (2)	2.7218 (15)	180 (2)
O2*W*—H4*W*···O2^ii^	0.86 (1)	1.87 (1)	2.7182 (15)	171 (2)
C10—H10*A*···O3^iii^	0.99	2.49	3.4253 (17)	158
C12—H12*A*···O2*W*^iv^	0.99	2.49	3.3711 (16)	147
C12—H12*B*···O2^v^	0.99	2.57	3.4818 (16)	153

Symmetry codes: (i) −*x*, *y*−1/2, −*z*+3/2; (ii) *x*−1, *y*+1, *z*; (iii) −*x*+2, −*y*+1, −*z*+1; (iv) *x*+1, *y*−1, *z*; (v) *x*−1, *y*, *z*.

### (2) 1,4-Diazabicyclo[2.2.2]octane-1,4-diium bis(4-nitrobenzoate) Crystal data

**Table:**

C_6_H_14_N_2_^2+^·2C_7_H_4_NO_4_^−^	*Z* = 2
*M**_r_* = 446.42	*F*(000) = 468
Triclinic, *P*1	*D*_x_ = 1.486 Mg m^−^^3^
Hall symbol: -P 1	Cu *K*α radiation, λ = 1.54184 Å
*a* = 9.1036 (4) Å	Cell parameters from 10186 reflections
*b* = 9.5027 (3) Å	θ = 3.8–76.8°
*c* = 12.0736 (3) Å	µ = 0.99 mm^−^^1^
α = 73.982 (3)°	*T* = 100 K
β = 83.624 (3)°	Prism, colourless
γ = 88.661 (3)°	0.40 × 0.40 × 0.20 mm
*V* = 997.68 (6) Å^3^	

### (2) 1,4-Diazabicyclo[2.2.2]octane-1,4-diium bis(4-nitrobenzoate) Data collection

**Table:**

Agilent SuperNova Dual diffractometer with an Atlas detector	4101 independent reflections
Radiation source: SuperNova (Cu) X-ray Source	3775 reflections with *I* > 2σ(*I*)
Mirror monochromator	*R*_int_ = 0.046
Detector resolution: 10.4041 pixels mm^-1^	θ_max_ = 75.0°, θ_min_ = 3.8°
ω scan	*h* = −11→11
Absorption correction: multi-scan (CrysAlis PRO; Agilent, 2013)	*k* = −11→11
*T*_min_ = 0.991, *T*_max_ = 1.000	*l* = −15→15
17849 measured reflections	

### (2) 1,4-Diazabicyclo[2.2.2]octane-1,4-diium bis(4-nitrobenzoate) Refinement

**Table:**

Refinement on *F*^2^	Primary atom site location: structure-invariant direct methods
Least-squares matrix: full	Secondary atom site location: difference Fourier map
*R*[*F*^2^ > 2σ(*F*^2^)] = 0.074	Hydrogen site location: inferred from neighbouring sites
*wR*(*F*^2^) = 0.229	H atoms treated by a mixture of independent and constrained refinement
*S* = 1.12	*w* = 1/[σ^2^(*F*_o_^2^) + (0.1192*P*)^2^ + 1.3867*P*] where *P* = (*F*_o_^2^ + 2*F*_c_^2^)/3
4101 reflections	(Δ/σ)_max_ < 0.001
295 parameters	Δρ_max_ = 0.60 e Å^−^^3^
2 restraints	Δρ_min_ = −0.58 e Å^−^^3^

### (2) 1,4-Diazabicyclo[2.2.2]octane-1,4-diium bis(4-nitrobenzoate) Special details

**Table:**

Geometry. All s.u.'s (except the s.u. in the dihedral angle between two l.s. planes) are estimated using the full covariance matrix. The cell s.u.'s are taken into account individually in the estimation of s.u.'s in distances, angles and torsion angles; correlations between s.u.'s in cell parameters are only used when they are defined by crystal symmetry. An approximate (isotropic) treatment of cell s.u.'s is used for estimating s.u.'s involving l.s. planes.
Refinement. Refinement of *F*^2^ against ALL reflections. The weighted *R*-factor *wR* and goodness of fit *S* are based on *F*^2^, conventional *R*-factors *R* are based on *F*, with *F* set to zero for negative *F*^2^. The threshold expression of *F*^2^ > 2σ(*F*^2^) is used only for calculating *R*-factors(gt) *etc*. and is not relevant to the choice of reflections for refinement. *R*-factors based on *F*^2^ are statistically about twice as large as those based on *F*, and *R*- factors based on ALL data will be even larger.

### (2) 1,4-Diazabicyclo[2.2.2]octane-1,4-diium bis(4-nitrobenzoate) Fractional atomic coordinates and isotropic or equivalent isotropic displacement parameters (Å^2^)

**Table:**

	*x*	*y*	*z*	*U*_iso_*/*U*_eq_	
O1	0.6213 (2)	0.7377 (2)	0.82692 (16)	0.0320 (5)	
O2	0.4577 (2)	0.9073 (2)	0.75460 (15)	0.0301 (4)	
O3	0.3598 (2)	0.7520 (2)	1.38056 (16)	0.0365 (5)	
O4	0.2049 (2)	0.9218 (2)	1.31087 (17)	0.0343 (5)	
N1	0.3033 (3)	0.8347 (2)	1.29939 (18)	0.0278 (5)	
C1	0.5159 (3)	0.8269 (3)	0.8358 (2)	0.0230 (5)	
C2	0.4601 (3)	0.8288 (3)	0.9588 (2)	0.0225 (5)	
C3	0.3361 (3)	0.9096 (3)	0.9783 (2)	0.0239 (5)	
H3A	0.2873	0.9646	0.9148	0.029*	
C4	0.2829 (3)	0.9105 (3)	1.0906 (2)	0.0245 (5)	
H4A	0.1976	0.9649	1.1051	0.029*	
C5	0.3578 (3)	0.8300 (3)	1.1807 (2)	0.0244 (5)	
C6	0.4829 (3)	0.7496 (3)	1.1642 (2)	0.0269 (5)	
H6	0.5320	0.6958	1.2280	0.032*	
C7	0.5343 (3)	0.7498 (3)	1.0515 (2)	0.0250 (5)	
H7	0.6202	0.6961	1.0374	0.030*	
O5	0.8758 (2)	0.7357 (2)	0.20757 (15)	0.0345 (5)	
O6	1.0354 (2)	0.5601 (2)	0.27470 (17)	0.0385 (5)	
O7	1.3135 (2)	0.6129 (2)	−0.29233 (18)	0.0368 (5)	
O8	1.1731 (2)	0.7994 (2)	−0.35307 (16)	0.0347 (5)	
N2	1.2191 (2)	0.7014 (3)	−0.27572 (19)	0.0287 (5)	
C8	0.9814 (3)	0.6492 (3)	0.1953 (2)	0.0256 (5)	
C9	1.0424 (3)	0.6604 (3)	0.0705 (2)	0.0233 (5)	
C10	1.1613 (3)	0.5752 (3)	0.0471 (2)	0.0253 (5)	
H10	1.2030	0.5075	0.1090	0.030*	
C11	1.2196 (3)	0.5889 (3)	−0.0670 (2)	0.0264 (5)	
H11	1.3010	0.5311	−0.0841	0.032*	
C12	1.1562 (3)	0.6885 (3)	−0.1550 (2)	0.0252 (5)	
C13	1.0376 (3)	0.7750 (3)	−0.1345 (2)	0.0259 (5)	
H13	0.9963	0.8426	−0.1967	0.031*	
C14	0.9806 (3)	0.7597 (3)	−0.0201 (2)	0.0255 (5)	
H14	0.8989	0.8174	−0.0034	0.031*	
N3	0.6982 (2)	0.7378 (2)	0.61824 (18)	0.0254 (5)	
H3N	0.665 (3)	0.734 (4)	0.6902 (12)	0.030*	
N4	0.7971 (2)	0.7223 (2)	0.41892 (18)	0.0261 (5)	
H4N	0.830 (3)	0.728 (4)	0.3454 (12)	0.031*	
C15	0.8607 (3)	0.7624 (3)	0.6003 (2)	0.0274 (5)	
H15A	0.9072	0.6984	0.6663	0.033*	
H15B	0.8832	0.8655	0.5954	0.033*	
C16	0.9224 (3)	0.7273 (3)	0.4870 (2)	0.0310 (6)	
H16A	0.9946	0.8035	0.4418	0.037*	
H16B	0.9733	0.6317	0.5047	0.037*	
C17	0.6672 (3)	0.5903 (3)	0.6066 (2)	0.0269 (5)	
H17A	0.5616	0.5648	0.6317	0.032*	
H17B	0.7276	0.5163	0.6564	0.032*	
C18	0.7046 (3)	0.5908 (3)	0.4791 (2)	0.0299 (6)	
H18A	0.7592	0.5009	0.4744	0.036*	
H18B	0.6127	0.5935	0.4418	0.036*	
C19	0.6263 (3)	0.8510 (3)	0.5300 (2)	0.0304 (6)	
H19A	0.6328	0.9477	0.5453	0.036*	
H19B	0.5206	0.8261	0.5334	0.036*	
C20	0.7063 (3)	0.8562 (3)	0.4098 (2)	0.0318 (6)	
H20A	0.6330	0.8614	0.3541	0.038*	
H20B	0.7704	0.9444	0.3814	0.038*	

### (2) 1,4-Diazabicyclo[2.2.2]octane-1,4-diium bis(4-nitrobenzoate) Atomic displacement parameters (Å^2^)

**Table:**

	*U*^11^	*U*^22^	*U*^33^	*U*^12^	*U*^13^	*U*^23^
O1	0.0368 (10)	0.0397 (11)	0.0185 (9)	0.0127 (8)	−0.0025 (7)	−0.0077 (7)
O2	0.0355 (10)	0.0357 (10)	0.0175 (8)	0.0069 (8)	−0.0028 (7)	−0.0053 (7)
O3	0.0476 (12)	0.0394 (11)	0.0182 (9)	0.0023 (9)	−0.0009 (8)	−0.0022 (8)
O4	0.0375 (11)	0.0371 (10)	0.0264 (9)	0.0021 (8)	0.0072 (8)	−0.0096 (8)
N1	0.0331 (12)	0.0299 (11)	0.0189 (10)	−0.0031 (9)	0.0022 (8)	−0.0060 (8)
C1	0.0243 (11)	0.0254 (12)	0.0183 (11)	−0.0019 (9)	−0.0008 (9)	−0.0047 (9)
C2	0.0255 (12)	0.0223 (11)	0.0190 (11)	−0.0027 (9)	−0.0012 (9)	−0.0045 (9)
C3	0.0255 (12)	0.0255 (12)	0.0198 (11)	−0.0014 (9)	−0.0041 (9)	−0.0041 (9)
C4	0.0249 (12)	0.0250 (12)	0.0229 (12)	−0.0006 (9)	0.0010 (9)	−0.0066 (9)
C5	0.0300 (12)	0.0256 (12)	0.0162 (11)	−0.0037 (10)	0.0026 (9)	−0.0050 (9)
C6	0.0317 (13)	0.0275 (12)	0.0187 (11)	0.0016 (10)	−0.0043 (10)	−0.0013 (9)
C7	0.0257 (12)	0.0250 (12)	0.0222 (12)	0.0019 (9)	0.0003 (9)	−0.0040 (9)
O5	0.0401 (11)	0.0445 (11)	0.0163 (9)	0.0144 (9)	−0.0005 (7)	−0.0062 (8)
O6	0.0454 (12)	0.0458 (12)	0.0193 (9)	0.0158 (9)	−0.0035 (8)	−0.0019 (8)
O7	0.0349 (10)	0.0484 (12)	0.0294 (10)	0.0019 (9)	0.0053 (8)	−0.0180 (9)
O8	0.0352 (10)	0.0478 (12)	0.0194 (9)	−0.0030 (9)	−0.0015 (7)	−0.0068 (8)
N2	0.0256 (11)	0.0382 (12)	0.0230 (11)	−0.0036 (9)	0.0010 (8)	−0.0105 (9)
C8	0.0264 (12)	0.0291 (12)	0.0192 (12)	0.0002 (10)	−0.0015 (9)	−0.0037 (9)
C9	0.0247 (12)	0.0265 (12)	0.0184 (11)	−0.0009 (9)	−0.0018 (9)	−0.0057 (9)
C10	0.0258 (12)	0.0245 (12)	0.0235 (12)	−0.0003 (9)	−0.0039 (10)	−0.0026 (9)
C11	0.0247 (12)	0.0264 (12)	0.0275 (13)	0.0005 (9)	0.0013 (10)	−0.0080 (10)
C12	0.0255 (12)	0.0298 (12)	0.0204 (12)	−0.0048 (10)	0.0015 (9)	−0.0081 (10)
C13	0.0272 (12)	0.0312 (13)	0.0180 (11)	−0.0008 (10)	−0.0035 (9)	−0.0043 (9)
C14	0.0243 (12)	0.0294 (12)	0.0215 (12)	0.0036 (9)	−0.0013 (9)	−0.0056 (10)
N3	0.0270 (11)	0.0308 (11)	0.0176 (10)	0.0066 (9)	−0.0021 (8)	−0.0061 (8)
N4	0.0290 (11)	0.0304 (11)	0.0169 (10)	0.0033 (9)	0.0004 (8)	−0.0045 (8)
C15	0.0289 (13)	0.0309 (13)	0.0228 (12)	0.0008 (10)	−0.0052 (10)	−0.0072 (10)
C16	0.0258 (12)	0.0425 (15)	0.0232 (12)	0.0004 (11)	0.0003 (10)	−0.0075 (11)
C17	0.0280 (12)	0.0312 (13)	0.0188 (12)	−0.0005 (10)	−0.0002 (9)	−0.0033 (9)
C18	0.0365 (14)	0.0306 (13)	0.0217 (12)	−0.0009 (11)	−0.0018 (10)	−0.0062 (10)
C19	0.0341 (14)	0.0339 (14)	0.0214 (12)	0.0121 (11)	−0.0040 (10)	−0.0056 (10)
C20	0.0408 (15)	0.0317 (13)	0.0195 (12)	0.0090 (11)	−0.0038 (10)	−0.0018 (10)

### (2) 1,4-Diazabicyclo[2.2.2]octane-1,4-diium bis(4-nitrobenzoate) Geometric parameters (Å, º)

**Table:**

O1—C1	1.281 (3)	C12—C13	1.384 (4)
O2—C1	1.228 (3)	C13—C14	1.390 (3)
O3—N1	1.229 (3)	C13—H13	0.9500
O4—N1	1.228 (3)	C14—H14	0.9500
N1—C5	1.475 (3)	N3—C17	1.483 (3)
C1—C2	1.520 (3)	N3—C15	1.485 (3)
C2—C3	1.386 (3)	N3—C19	1.487 (3)
C2—C7	1.398 (3)	N3—H3N	0.879 (10)
C3—C4	1.390 (3)	N4—C20	1.486 (3)
C3—H3A	0.9500	N4—C16	1.487 (3)
C4—C5	1.384 (4)	N4—C18	1.487 (3)
C4—H4A	0.9500	N4—H4N	0.890 (10)
C5—C6	1.383 (4)	C15—C16	1.539 (3)
C6—C7	1.388 (3)	C15—H15A	0.9900
C6—H6	0.9500	C15—H15B	0.9900
C7—H7	0.9500	C16—H16A	0.9900
O5—C8	1.273 (3)	C16—H16B	0.9900
O6—C8	1.231 (3)	C17—C18	1.538 (3)
O7—N2	1.228 (3)	C17—H17A	0.9900
O8—N2	1.226 (3)	C17—H17B	0.9900
N2—C12	1.479 (3)	C18—H18A	0.9900
C8—C9	1.523 (3)	C18—H18B	0.9900
C9—C10	1.387 (3)	C19—C20	1.538 (3)
C9—C14	1.395 (3)	C19—H19A	0.9900
C10—C11	1.393 (4)	C19—H19B	0.9900
C10—H10	0.9500	C20—H20A	0.9900
C11—C12	1.382 (4)	C20—H20B	0.9900
C11—H11	0.9500		
			
O4—N1—O3	124.0 (2)	C17—N3—C15	108.68 (19)
O4—N1—C5	117.9 (2)	C17—N3—C19	109.7 (2)
O3—N1—C5	118.0 (2)	C15—N3—C19	109.7 (2)
O2—C1—O1	125.6 (2)	C17—N3—H3N	105 (2)
O2—C1—C2	119.0 (2)	C15—N3—H3N	110 (2)
O1—C1—C2	115.4 (2)	C19—N3—H3N	114 (2)
C3—C2—C7	120.3 (2)	C20—N4—C16	109.6 (2)
C3—C2—C1	119.7 (2)	C20—N4—C18	109.5 (2)
C7—C2—C1	120.0 (2)	C16—N4—C18	108.9 (2)
C2—C3—C4	120.2 (2)	C20—N4—H4N	103 (2)
C2—C3—H3A	119.9	C16—N4—H4N	111 (2)
C4—C3—H3A	119.9	C18—N4—H4N	115 (2)
C5—C4—C3	118.2 (2)	N3—C15—C16	108.8 (2)
C5—C4—H4A	120.9	N3—C15—H15A	109.9
C3—C4—H4A	120.9	C16—C15—H15A	109.9
C6—C5—C4	123.2 (2)	N3—C15—H15B	109.9
C6—C5—N1	118.9 (2)	C16—C15—H15B	109.9
C4—C5—N1	117.9 (2)	H15A—C15—H15B	108.3
C5—C6—C7	117.9 (2)	N4—C16—C15	108.4 (2)
C5—C6—H6	121.1	N4—C16—H16A	110.0
C7—C6—H6	121.1	C15—C16—H16A	110.0
C6—C7—C2	120.3 (2)	N4—C16—H16B	110.0
C6—C7—H7	119.8	C15—C16—H16B	110.0
C2—C7—H7	119.8	H16A—C16—H16B	108.4
O8—N2—O7	124.1 (2)	N3—C17—C18	109.0 (2)
O8—N2—C12	118.0 (2)	N3—C17—H17A	109.9
O7—N2—C12	118.0 (2)	C18—C17—H17A	109.9
O6—C8—O5	125.5 (2)	N3—C17—H17B	109.9
O6—C8—C9	119.2 (2)	C18—C17—H17B	109.9
O5—C8—C9	115.3 (2)	H17A—C17—H17B	108.3
C10—C9—C14	120.2 (2)	N4—C18—C17	108.3 (2)
C10—C9—C8	120.2 (2)	N4—C18—H18A	110.0
C14—C9—C8	119.6 (2)	C17—C18—H18A	110.0
C9—C10—C11	120.0 (2)	N4—C18—H18B	110.0
C9—C10—H10	120.0	C17—C18—H18B	110.0
C11—C10—H10	120.0	H18A—C18—H18B	108.4
C12—C11—C10	118.5 (2)	N3—C19—C20	108.2 (2)
C12—C11—H11	120.8	N3—C19—H19A	110.1
C10—C11—H11	120.8	C20—C19—H19A	110.1
C11—C12—C13	122.9 (2)	N3—C19—H19B	110.1
C11—C12—N2	117.8 (2)	C20—C19—H19B	110.1
C13—C12—N2	119.3 (2)	H19A—C19—H19B	108.4
C12—C13—C14	117.9 (2)	N4—C20—C19	109.0 (2)
C12—C13—H13	121.1	N4—C20—H20A	109.9
C14—C13—H13	121.1	C19—C20—H20A	109.9
C13—C14—C9	120.5 (2)	N4—C20—H20B	109.9
C13—C14—H14	119.7	C19—C20—H20B	109.9
C9—C14—H14	119.7	H20A—C20—H20B	108.3
			
O2—C1—C2—C3	6.3 (4)	C10—C11—C12—N2	−179.8 (2)
O1—C1—C2—C3	−173.0 (2)	O8—N2—C12—C11	−172.9 (2)
O2—C1—C2—C7	−173.4 (2)	O7—N2—C12—C11	7.4 (3)
O1—C1—C2—C7	7.3 (3)	O8—N2—C12—C13	7.4 (3)
C7—C2—C3—C4	−1.2 (4)	O7—N2—C12—C13	−172.3 (2)
C1—C2—C3—C4	179.1 (2)	C11—C12—C13—C14	−0.1 (4)
C2—C3—C4—C5	0.5 (4)	N2—C12—C13—C14	179.7 (2)
C3—C4—C5—C6	0.3 (4)	C12—C13—C14—C9	0.2 (4)
C3—C4—C5—N1	178.4 (2)	C10—C9—C14—C13	−0.2 (4)
O4—N1—C5—C6	170.3 (2)	C8—C9—C14—C13	178.0 (2)
O3—N1—C5—C6	−9.1 (4)	C17—N3—C15—C16	50.7 (3)
O4—N1—C5—C4	−7.9 (3)	C19—N3—C15—C16	−69.2 (3)
O3—N1—C5—C4	172.7 (2)	C20—N4—C16—C15	50.2 (3)
C4—C5—C6—C7	−0.3 (4)	C18—N4—C16—C15	−69.6 (3)
N1—C5—C6—C7	−178.4 (2)	N3—C15—C16—N4	15.8 (3)
C5—C6—C7—C2	−0.4 (4)	C15—N3—C17—C18	−69.4 (2)
C3—C2—C7—C6	1.1 (4)	C19—N3—C17—C18	50.5 (3)
C1—C2—C7—C6	−179.2 (2)	C20—N4—C18—C17	−68.8 (3)
O6—C8—C9—C10	−2.6 (4)	C16—N4—C18—C17	51.1 (3)
O5—C8—C9—C10	177.5 (2)	N3—C17—C18—N4	15.8 (3)
O6—C8—C9—C14	179.2 (2)	C17—N3—C19—C20	−68.8 (3)
O5—C8—C9—C14	−0.7 (4)	C15—N3—C19—C20	50.6 (3)
C14—C9—C10—C11	0.1 (4)	C16—N4—C20—C19	−68.9 (3)
C8—C9—C10—C11	−178.1 (2)	C18—N4—C20—C19	50.5 (3)
C9—C10—C11—C12	0.0 (4)	N3—C19—C20—N4	15.6 (3)
C10—C11—C12—C13	0.0 (4)		

### (2) 1,4-Diazabicyclo[2.2.2]octane-1,4-diium bis(4-nitrobenzoate) Hydrogen-bond geometry (Å, º)

**Table:**

*D*—H···*A*	*D*—H	H···*A*	*D*···*A*	*D*—H···*A*
N3—H3*N*···O1	0.88 (2)	1.66 (2)	2.539 (3)	173 (2)
N4—H4*N*···O5	0.89 (2)	1.65 (2)	2.542 (3)	175 (3)
C15—H15*A*···O6^i^	0.99	2.42	3.193 (3)	134
C16—H16*A*···O4^ii^	0.99	2.42	3.375 (3)	161
C17—H17*A*···O7^iii^	0.99	2.42	3.338 (3)	153
C17—H17*B*···O6^i^	0.99	2.42	3.313 (3)	149
C20—H20*A*···O2^iv^	0.99	2.41	3.043 (3)	121
C20—H20*B*···O8^v^	0.99	2.42	3.339 (3)	154

Symmetry codes: (i) −*x*+2, −*y*+1, −*z*+1; (ii) *x*+1, *y*, *z*−1; (iii) *x*−1, *y*, *z*+1; (iv) −*x*+1, −*y*+2, −*z*+1; (v) −*x*+2, −*y*+2, −*z*.
